# Efficacy of Angiotensin Converting Enzyme Inhibitors and Angiotensin Receptor-Neprilysin Inhibitors in the Treatment of Chronic Heart Failure: A Review of Landmark Trials

**DOI:** 10.7759/cureus.3913

**Published:** 2019-01-19

**Authors:** Sosipatros Bratsos

**Affiliations:** 1 Internal Medicine, Imperial College London, London, GBR

**Keywords:** heart failure, ace inhibitor, enalapril, angiotensin receptor–neprilysin inhibitor, sacubitril/valsartan

## Abstract

Heart failure (HF) is a multi-faceted clinical condition affecting up to 2% of the population in the developed world and is linked to significant morbidity and mortality, therefore posing a major public health concern. To this date, pharmacotherapy for HF has mainly focused on chronic HF with reduced ejection fraction (HFrEF), with angiotensin converting enzyme inhibitors (ACEi) being at the centre of the management plan, alongside angiotensin-receptor-blockers (ARBs), β-blockers (BB) and mineralocorticoid receptor antagonists (MRAs). A novel and recently approved therapy, however, involving angiotensin receptor–neprilysin inhibitors (ARNI), has shown very promising results and comparable to those of ACEi, which raises the question of whether ACEi should remain the first-line treatment option for HFrEF. In this review, the evidence regarding the clinical efficacy of ACEi and ARNI in the treatment of HFrEF is discussed, with emphasis placed on the major landmark trials.

## Introduction and background

Heart failure (HF) is a complex clinical syndrome that can result from either structural or functional cardiac abnormalities, compromising the ability of the ventricles to fill and / or eject blood [1]. The heart is hence unable to generate sufficient cardiac output to meet the metabolic demands of tissues, causing symptoms like dyspnoea and fatigue, and signs such as elevated jugular venous pressure, tachycardia, or peripheral oedema [2]. HF can be classified according to the severity of the patient’s symptoms via the New York Heart Association (NYHA), which is depicted below (Table 1) [1]. It poses a major and growing public health concern, affecting 1%-2% of the population in developed countries, with the prevalence rising to more than 10% in those aged 70 or more. Despite advances in treatment, HF is associated with significant morbidity and mortality (five-year survival rate is 50%) and is responsible for substantial healthcare costs ($39 billion per annum in the US) [3-4]. Pharmacotherapy for HF that is linked to improved morbidity or mortality currently includes drugs such as angiotensin-converting enzyme inhibitors (ACEi), angiotensin receptor blockers (ARBs), β-blockers (BB) and mineralocorticoid receptor antagonists (MRAs), while several other drugs with promising benefits are under development. So far, most drugs demonstrating beneficial outcomes in clinical trials have been tested in patients with chronic HF with reduced ejection fraction (HFrEF) (defined as ejection fraction <40% of normal) [5]. The cornerstone and first-line treatment option for chronic HFrEF currently involves ACEi, but a newly licensed angiotensin receptor-neprilysin inhibitor (ARNI) has recently been recommended as a replacement for ACEi in patients with HFrEF NYHA II-IV [6]. Therefore, in this paper, the efficacy of enalapril, an ACEi, is discussed in the treatment of chronic HFrEF, and then compared to the efficacy of sacubitril/valsartan, an ARNI. The aforementioned drugs were chosen as representative of their respective drug classes, due to the amount and quality of literature present, which also allows for a direct, head to head comparison.

**Table 1:** New York Heart Association (NYHA) Functional Classification The New York Heart Association Functional classification system for heart failure ranges from class I, where patients essentially have no symptoms of heart failure, to class IV, where patients experience symptoms of heart failure even at rest. The symptoms include fatigue, palpitations and dyspnoea [1].

Class	Symptom Severity
I	Symptoms of heart failure only at levels that would limit normal individuals
II	Symptoms of heart failure on ordinary exertion
III	Symptoms of heart failure on less-than-ordinary exertion
IV	Symptoms of heart failure at rest

## Review

Mechanism of action

Ace Inhibitors

ACEi have been shown in many studies to attenuate ventricular remodelling and improve ventricular function in patients with HF [7]. This reverse-remodelling can be explained by several proposed mechanisms. Specifically, ACEi have a profound effect on the neuro-hormonal state of patients with HF through their interference with the renin-angiotensin-aldosterone system (RAAS), via the inhibition of the conversion of angiotensin I to angiotensin II. Decreased levels of angiotensin II enhance natriuresis and lower blood pressure (BP), by reducing sympathetic activity, aldosterone and vasopressin release and thus vasoconstriction. In addition, ACEi prevent the breakdown of bradykinin, thus inducing vasodilation and further BP reduction [8]. Lowered arterial and venous pressure in turn leads to reduced preload and importantly afterload, which results in increased stroke volume and improved ejection fraction. ACEi can also inhibit ventricular remodelling by actions at a cellular level, specifically by limiting cardiac hypertrophy and myocardial fibrosis, while also attenuating cardiomyocyte apoptosis. In these ways, ACEi have been shown to have beneficial effects in chronic HF [4, 9, 10].

Angiotensin Receptor–Neprilysin Inhibitors

Sacubitril/valsartan is a combination drug that uses an ARB (valsartan) plus a neprilysin inhibitor (sacubitril) in a one:one molar ratio. Valsartan is an angiotensin type I receptors (AT1)-inhibitor, thus causing vasodilation, reduced aldosterone production, increased nartiuresis and therefore reduced BP. Sacubitril inhibits neprilysin, which is an endopeptidase responsible for deactivating active natriuretic peptides. Thus, blocking this enzyme results in enhanced levels of natriuretic peptides, such as BNP, bradykinin, and adrenomedullin, which result in increased generation of myocardial cyclic guanosine monophosphate (cGMP) and therefore improved myocardial relaxation and reduced hypertrophy, which collectively oppose the overstimulated neurohormonal state of HF patients [11]. This leads to reduced vasoconstriction, sodium retention and adverse ventricular remodelling, potentially producing favourable clinical outcomes in patients with HF [12].

Efficacy

The Cooperative North Scandinavian Enalapril Survival Study (CONSENSUS) trial was the first landmark paper to evaluate the effect of enalapril on mortality, compared to placebo, in patients with severe congestive HFrEF [13]. It was shown that the six and 12-month mortality rates were 40% (P=0.002) and 31% (P=0.001) lower in the enalapril group compared to placebo, respectively. By the end of the study, there was a 27% (P=0.003) reduction in mortality in the enalapril vs placebo group (68 vs. 50) and importantly, a 50% reduction in mortality due to the progression of HF in the enalapril-treated group (P<0.001). The numbers and causes of death in the respective treatment groups in the CONSENSUS trial are summarized below (Table 2). Despite that, no significant improvement in mortality rate was observed in patients receiving vasodilators at baseline (P=0.11), suggesting that the added benefit of enalapril compared to vasodilator therapy is limited. In addition, 22% of the placebo-treated patients and 42% of the enalapril-treated patients had improvement in their NYHA classification (P<0.001). In fact, change of NYHA class IV to class I or II was observed in 16 patients in the enalapril group vs two in the placebo. However, the validity of the NYHA classification in terms of characterizing HF progression has been questioned due to inconsistent results and high inter-operator variability [14]. Another benefit was the reduction in heart size (P=0.02) (final heart sizes in the two groups were similar, however) and the concomitant use of other cardiovascular drugs in the enalapril compared to the placebo-treated patients.

**Table 2:** CONSENSUS trial: Number and causes of deaths, according to treatment group The number of deaths and their causes in the CONSENSUS trial according to the treatment received are shown. There was a statistically significant reduction in any cardiac death (P=0.001), death due to the progression of congestive heart failure (P=0.001) and total mortality (P=0.003) in patients treated with enalapril compared to placebo. No difference between the groups was found in sudden cardiac death rates (P>0.25), however [13].

Cause of death	Placebo (n=126)	Enalapril (n=127)	Risk reduction (%)	P value
Any cardiac death	64	44	31	0.001
Sudden cardiac death	14	14	0	>0.25
Worsening of heart failure	44	22	50	0.001
Total mortality	68	50	27	0.003

In this double-blind, placebo-controlled, parallel-group trial, 253 patients were randomized to minimize selection bias and confounding, by equally allocating covariates affecting treatment outcomes between the groups. However, ethnicity data were not provided, and the selected patients had a higher prevalence of atrial fibrillation compared to most patients with NYHA IV HF and thus the results may not be representative of the target population [15-16]. Moreover, since only patients with NYHA IV were randomised, it is unclear whether similar benefits might be realized by less severely impaired patients with HF. Furthermore, the limited follow-up due to the premature termination of the trial did not allow the examination of the duration of these benefits. Also, although the survival benefits of enalapril were essentially solely attributed to the decrease in progression of HF, with no effect on of sudden cardiac death rates, many studies have emphasized the difficulty of determining the mode of death in patients with HF and thus the exact cause of death is debatable [17]. Hence, it is likely that the improvement in mortality rates was not related to the improvement in HF progression, but to a decrease in sudden death rates. Another limitation was that no specific mechanism was identified for the beneficial effects of enalapril.

The Studies of Left Ventricular Dysfunction trial (SOLVD-T; treatment arm), looked at the effect of enalapril on mortality and hospitalizations for congestive HFrEF in 2569 patients [18]. After four years, there was a 16% reduction in the cumulative mortality rate in the enalapril compared to the placebo-treated group (P=0.0036). Notably, the greatest difference in mortality was in deaths due to progressive HF (risk reduction 22%; P<0.0045). In addition, 57% of patients in the placebo group died or were hospitalized for worsening congestive HF, as compared with 48% in the enalapril group (risk reduction 26%; P<0.0001). The number of deaths, their causes, and the number of hospitalizations for HF between the treatment groups in the SOLV-D trial are summarised below (Table 3). It was estimated that treating 1000 patients with congestive HF (similar to those in this study) with enalapril for three years would prevent 50 premature deaths and 350 hospitalizations. Moreover, the benefits of enalapril were likely to be underestimated, as more patients taking placebo compared to enalapril received other vasodilators during the trial for worsening HF. It was hypothesised that the reductions in deaths and hospitalization rates may have been due to improvements in ejection fraction and exercise capacity and decreased symptoms of congestion, attributed to the decreased preload and afterload by enalapril.

**Table 3:** Studies of Left Ventricular Dysfunction (SOLVD) trial: Number and causes of deaths, according to treatment group Statistically significant reductions were achieved in the SOLVD trial in the total number of deaths (P<0.0036), deaths or hospitalisations for heart failure (P<0.001), cardiovascular deaths (P<0.002) and deaths due to worsening heart failure (P<0.0045) in patients receiving enalapril vs placebo [18].

Variable	Placebo (n=1284)	Enalapril (n=1285)	Risk reduction (%)	P value
Total deaths	39.7	35.2	16	<0.0036
Deaths or hospitalisations for Congestive Heart Failure	57.3	47.7	26	<0.0001
Cardiovascular deaths	35.9	31.1	18	<0.002
Deaths due to worsening heart failure	19.5	16.3	22	<0.0045

This well-randomized, double-blind, multi-centre and placebo-controlled study recruited more subjects that were followed-up over a longer period (average 41 months) than the CONSENSUS and other similar trials. It also included a broader range of patients with HF (NYHA I-IV) and examined both mortality and hospitalisation rates. However, most ethnicities and the female sex were under-represented (80% white and 80% male patients), giving rise to selection bias. As with CONSENSUS, the greatest treatment benefit was reduced deaths from progressive HF, but in patients with class IV HF, there was no difference in mortality rate between the groups in SOLVD as opposed to CONSENSUS. This may have been due to chance, however, because of the small numbers of patients in this subgroup. Furthermore, the prognostic benefit was confined to patients with severe compromise, i.e. left ventricular ejection fraction (LVEF)<30%, although the limited efficacy in patients with 30%<LVEF<35% may have been due to the lack of power of the study, given the low mortality rate in this subgroup. Another important realisation is that although the commonest cause of death was worsening HF, it only accounted for less than half of total deaths, so that even a substantial reduction in the risk of this category would lead to a limited reduction in total mortality. As such, it is essential that more treatment strategies are explored for a significant overall mortality benefit to be achieved in these patients.

The Prospective Comparison of ARNI with ACEI to Determine Impact on Global Mortality and Morbidity in Heart Failure (PARADIGM-HF) trial compared the effects of sacubitril/valsartan, a now licensed ARNI, to enalapril, on cardiovascular mortality and hospitalisations for HF [19]. 8399 patients with HFrEF NYHA II-IV were randomly assigned to receive either sacubitril/valsartan or enalapril, and followed-up for a median of 27 months. Deaths due to cardiovascular causes were 13.3% in the sacubitril/valsartan group compared to 16.5% in the enalapril group (hazard ratio (HR), 0.80; P<0.001). Hospitalizations for HF were 12.8% and 15.6% of patients receiving sacubitril/valsartan and enalapril respectively (HR, 0.79; P<0.001). In addition, the benefit with respect to cardiovascular mortality was consistent irrespective of age, sex, ethnicity, ejection fraction and NYHA class, with no impact on the incidence of non-cardiovascular death, meaning that the mortality benefit was due to decreased cardiovascular risk. Further analysis revealed that the hazard for both sudden death (HR 0.80; P = 0.008) and death due to worsening HF (HR 0.79; P=0.034) was significantly reduced by treatment with sacubitril/valsartan [20]. A reflection of this are the lower levels of N-terminal pro b-type natriuretic peptide (NTproBNP) in the sacubitril/valsartan group, indicating reduced cardiac wall stress (NTproBNP, unlike B-type natriuretic peptide (BNP), is not a substrate for neprilysin and thus its lower levels did not reflect the expected action of the drug, rather the drug’s effect on the heart) [21]. Finally, sacubitril/valsartan significantly reduced all-cause mortality compared to enalapril (17.0% vs 19.8%, P<0.001) and this is also supported by a network meta-analysis comparing the efficacy of drugs and their combinations regarding all-cause mortality in patients with HFrEF, which showed that the combination of ARNI with BB and MRA resulted in greater overall mortality reduction than the combination of ACEI with BB and MRA [22]. In summary, sacubitril/valsartan was found to be superior to enalapril in reducing the risks of all-cause or cardiovascular mortality (including due to worsening HF) and for hospitalization for HF. Arguably, since ARBs and ACEi have been shown to produce comparable benefits in HF patients, the observed benefit in PARADIGM-HF is likely to be related to the added neprilysin inhibition [4]. The number of deaths from cardiovascular causes and hospitalisations for worsening HF in the PARADIGM-HF trial are depicted below in Table 4.

**Table 4:** PARADIGM-HF trial: Cardiovascular deaths and first hospitalisations from worsening heart failure Statistically significant reductions in death from cardiovascular causes (P<0.001) and first hospitalization for worsening heart failure (P<0.001) were achieved in patients treated with sacubitril/valsartan compared to enalapril in the PARADIGM-HF trial [19]. *PARADIGM - Prospective Comparison of ARNI with ACEI to Determine Impact on Global Mortality and Morbidity in Heart Failure

Variable	Sacubitril/valsartan (n=4187)	Enalapril (n=4212)	Hazard ratio	P-value
Cardiovascular deaths	13.3	16.5	0.80	<0.001
First hospitalisation for worsening heart failure	12.8	15.6	0.79	<0.001

Despite the statistically and clinically significant findings and its robust design (large, randomized, double-blind, parallel group, active-controlled) this trial had a few limitations. Firstly, the average population age (63.8) was lower than what would be observed in clinical practice, at least in the UK, while patients that were female, black or had shortened expected survival were under-represented, limiting the generalisability of the findings [23]. Also, only 15% and 7% of patients had received implantable cardioverter-defibrillator or cardiac resynchronisation therapy respectively prior to randomisation, possibly reflecting suboptimal background therapy [6]. Added to that, over half of patients on enalapril were not on MRAs, which may have caused unopposed aldosterone escape and thus weakened anti-RAAS activity [24]. On the other hand, an ACEi-MRA combination does not necessarily reflect optimal therapy. Effective application of background therapy is vital in order to accurately evaluate a potential replacement in the cornerstone of HF therapy, i.e. ACEi. Another issue was that the dose of the two drugs were not comparable. The maximum tolerated dose of valsartan (320 mg) in addition to neprilysin was given compared to moderate dose of enalapril (20 mg). Nevertheless, it was stated by the authors that the enalapril dose was based on previous studies assessing its efficacy, e.g. SOLVD, but in practice doses up to 40 mg can be prescribed [25-26]. This could perhaps explain the decreased mean systolic blood pressure (BP) (-2.7 mmHg) in the enalapril vs the sacubitril/valsartan group, although this could also be due to the added neprilysin inhibition. Despite that, the benefit of sacubitril/valsartan over enalapril was not explained by the BP difference. Furthermore, substituting enalapril for pre-randomisation physician-selected ACEi may not have ensured dose equivalence and thus adequate RAAS inhibition, which was reflected in that significant decreases in cardiovascular mortality or hospitalisations for HF in favour of sacubitril/valsartan were seen only in patients who were on ACEi pre-randomization. Significant differences were not observed in those previously not on ACEi (and therefore not subject to reduced RAAS inhibition), however. Finally, Novartis was the sponsor and employer of many of the study’s authors, which could raise the suspicion of industry bias.

## Conclusions

HF is a significant public health problem that causes a tremendous financial burden on the healthcare system. Its rising incidence and ominous prognosis necessitate the implementation of effective management. In this review, the mechanism of action and efficacy of enalapril in relation to HFrEF treatment was discussed and compared to sacubitril/valsartan. ACEi have been a fundamental constituent of HF management for many years, following the results of landmark studies, such as the CONSENSUS and SOLVD, where enalapril was shown to produce significant reductions in all-cause and cardiovascular mortality as well as hospitalizations for HF compared to placebo. Recently, however, sacubitril/valsartan, which belongs to a new class of drugs, i.e. ARNI, was shown in the PARADIGM-HF trial to be superior to enalapril in reducing cardiovascular mortality and HF hospitalisations, which led to its approval by Europe and the US as an alternative for ACEi in patients with HFrEF NYHA II-IV. However, more and longer-term trials might be necessary to conclusively compare the efficacy of two drugs, as well as their safety, which despite being beyond the scope of this review, is a crucial component to evaluate. Moreover, the results of the above trials should be taken with caution, as several limitations were identified, which may affect their generalizability and applicability in real-life clinical practice. Finally, there is an inherent difficulty in assessing the comparative efficacy of single pharmaceutical agents, as usually a combination of several drugs is prescribed to most HF patients and few head-to-head trials exist, which is an issue that might need to be addressed in the future.
